# Poor Maternal Diet During Gestation Alters Offspring Muscle Morphometrics, Collagen Gene Expression, and Meat Tenderness in Sheep

**DOI:** 10.3390/ani16030486

**Published:** 2026-02-04

**Authors:** Mia Y. Kawaida, Amanda S. Reiter, Nicole M. Tillquist, Brandon I. Smith, Daniela A. Alambarrio, John M. Gonzalez, Stephanie N. Royko, Michela A. Brown, Shawn R. Re, Kristen E. Govoni, Steven A. Zinn, Sarah A. Reed

**Affiliations:** 1 Department of Animal Science, University of Connecticut, Storrs, CT 06269, USA; 2 Department of Animal Science, University of Georgia, Athens, GA 30602, USAjohngonz@uga.edu (J.M.G.)

**Keywords:** collagen, maternal nutrition, meat, muscle, sheep, tenderness

## Abstract

We hypothesized that maternal overfeeding and restricted feeding during gestation would alter the collagen content, muscle fiber cross-sectional area (CSA), and meat tenderness in offspring. Pregnant ewes were fed 100% (CON), 60% (RES), or 140% (OVER) of their requirements from day 30 of gestation until parturition. Male offspring were necropsied at 282 ± 1.8 days of age. Gene expression and CSA were quantified in the longissimus (LM) and semitendinosus (STN) muscles. The Warner–Bratzler shear force (WBSF) was quantified in LM. Data were analyzed by one-way ANOVA, with diet as a fixed effect. Differences were considered significant at *p* ≤ 0.05 or a tendency at *p* ≤ 0.10. Semitendinosus CSA was smaller in OVER and RES than CON rams (*p* = 0.02). Longissimus CSA was larger in RES than OVER and CON rams (*p* = 0.002). OVER LM had reduced WBSF compared with CON rams (*p* = 0.03). Myogenic genes *bone morphogenic protein 1* (BMP1) and *paired box 7* were greater in RES LM than OVER (*p* ≤ 0.02). Maternal diet altered the fibrogenic genes *fibronectin 1* (*p* = 0.07), *lysyl oxidase* (*p* = 0.07), and *collagen 1A1* (COL1A1; *p* = 0.08) in the LM and *COL1A1*, *COL3A1*, and *BMP1* (*p* ≤ 0.09) in the STN. Poor maternal diet during gestation alters muscle and meat characteristics that may impact meat quality.

## Simple Summary

Poor maternal nutrition during gestation alters the muscle growth of offspring, but how that impacts meat characteristics and quality is not well defined. Male offspring of ewes fed a restricted-fed diet had a greater longissimus muscle fiber size but reduced semitendinosus muscle fiber size, with no differences in longissimus or semitendinosus muscle weight. Offspring of ewes fed an overfed diet had loin chops that were more tender than offspring of restricted- or control-fed ewes. There were no differences in back fat or longissimus muscle collagen content as a result of maternal diet. However, gene expression related to muscle and collagen formation, and epigenetic markers, were altered as a result of maternal diet. These results suggest that a poor maternal diet impacts the meat characteristics that may alter the meat quality of sheep.

## 1. Introduction

In livestock, poor maternal nutrition during gestation causes altered pre- and postnatal growth, poor body composition (increased fat, reduced muscle), metabolic disorders, and organ dysfunction, all of which lead to poor health and a reduced quantity and quality of meat and milk products [1,2]. Poor maternal nutrition can be caused by an increased or decreased availability of total nutrients, energy, macronutrients, or micronutrients, with similar phenotypic outcomes [3]. Specifically, maternal restricted- and over-nutrition during gestation in ewes resulted in a reduced lean-to-fat ratio [4], reduced muscle fiber cross-sectional area [5,6], and increased fat deposition [6,7] in offspring. Further, both nutrient restriction and overfeeding reduced muscle fiber CSA growth in lambs from birth to 3 months of age [6]. Maternal nutrient restriction during early or late gestation resulted in fewer muscle fibers in late gestation [8] and an increased number of glycolytic myofibers in 8-month-old offspring [4]. Fetal muscle (gestational day 135) in lambs from obese ewes had a decreased primary muscle fiber diameter and increased collagen content [9], which persisted into adulthood [10,11]. Overall, these alterations in CSA, collagen content, and fiber type can negatively impact meat tenderness [12,13].

Adipogenesis, fibrogenesis, and myogenesis are critical in lipid accumulation, collagen deposition and crosslinking formation, and fetal muscle development, respectively, and can affect product quality aspects in livestock species [14,15]. Impairment of one or more of these processes can alter the composition of meat and may reduce meat quality [16]. Nutrient restriction during gestation, for instance, delayed adipose tissue development via the downregulation of mRNA expression related to adipogenesis, such as *CCAAT/enhancer-binding protein α (CEBPα)* and *peroxisome proliferator-activated receptor γ* (*PPARγ*) in Wagyu offspring [17], which may negatively impact meat flavor. Piglets that experienced nutrient deficiency due to improper placental nutrient delivery demonstrated decreased birthweight and greater collagen content in skeletal muscle [18], reducing meat tenderness. In contrast, progeny of first-calf dams fed 80% of metabolizable protein requirements during gestation had similar hot carcass weights, back fat thickness, and longissimus (LM) area to controls [19]. Similarly, overfeeding Ile de France dams during the last third of gestation did not affect the carcass weight, dressing percentage, commercial cut yields, loin-eye area, or shear force in male offspring at approximately 120 days of age [20]. However, the effects of poor maternal nutrition on carcass traits in sheep, especially over 8 months of age, are not well characterized. Therefore, the objective of this study was to determine the effects of poor maternal nutrition during gestation on postnatal muscle development and meat quality in ram offspring. We hypothesized that overfeeding and restricted feeding during gestation would negatively impact aspects of postnatal muscle growth and meat characteristics by altering muscle fiber size, collagen crosslinking, and adipogenic, fibrogenic, and/or myogenic gene expression.

## 2. Materials and Methods

### 2.1. Animals and Sample Collection

As previously described [21], multiparous pregnant Dorset ewes (n = 46) were individually fed one of three treatment diets: control-fed (100%; CON, n = 11), restricted-fed (60%; RES, n = 17), or overfed (140%; OVER, n = 15) based on the National Research Council (NRC) requirements for total digestible nutrients (TDN) for ewes carrying twins, starting at d 30 of gestation until parturition (Supplemental Table S1). Lambs (CON, n = 22; RES, n = 34; OVER, n = 30) were maintained with their dams in groups and fed creep feed (ad libitum, Home Fresh 18 Sheep Starter, Blue Seal, Litchfield, CT, USA) and second-cutting hay (ad libitum) until weaning. After weaning at 60 days of age, lambs were group-housed and fed grower feed (Home Fresh Shepherd 16, Blue Seal) to meet 100% of the NRC requirements. Due to reasons unrelated to the experiment, nine offspring did not complete the study (CON, n = 3; RES, n = 4; OVER, n = 2; not included in the figures or statistical analysis). At d 282 ± 1.82 of age, all ram lambs (CON = 12, RES = 18, OVER = 13) were euthanized by intravenous injection of Euthasol (0.22 mL/kg BW; Virbac, Fort Worth, TX, USA) containing 390 mg/mL of sodium pentobarbital and 50 mg/mL of sodium phenytoin. The backfat thickness and rib eye area (REA) were measured between the 12th and 13th ribs by using a grid specific to pork and lamb (Iowa State University Extension and Outreach, Ames, IA, USA). The LM was collected and weighed. A 5.08 cm loin roast was removed from between the 11th and 13th ribs, vacuum packaged, and aged for 10 d at 4 °C. After aging, the roasts were frozen at −20 °C. Portions of the LM adjacent to the loin sample, as well as portions of the semitendinosus (STN) muscle, were snap frozen in liquid nitrogen or embedded in Tissue-Tek OCT (Thermo Fisher Scientific, Waltham, MA, USA) and frozen in dry ice-cooled isopentane. A complete description of the experimental design, animals, and diets used was previously reported in [21].

### 2.2. Immunohistochemistry

Muscle fiber CSA and the lipid content were determined as previously described [6]. Briefly, LM and STN muscle samples were sectioned at 10 µm using a Microm HM 525 cryostat (Thermo Fisher Scientific), heat-fixed for 1 min, and fixed in 4% paraformaldehyde in phosphate-buffered saline (PBS) for 25 min followed by three 5 min PBS washes. The sections were incubated with wheat germ agglutinin (WGA; 1:400, Invitrogen, Waltham, MA, USA) in PBS for 1 h in the dark in a humidified box. Following three 5 min PBS washes, the slides were cover-slipped with a 1:9 PBS/glycerol solution. Slides were imaged using an AxioCam camera and AxioObserver microscope (Zeiss, Inc., Jena, Germany), with 5 to 10 images taken per muscle. ImageJ v. 1.54 (National Institutes of Health, Bethesda, MD, USA) was used to false-color the images. The cross-sectional area was measured as the region within the fiber boundary by using the area measurement tool in ImageJ. A minimum of 500 fibers per muscle were analyzed from at least 10 images from 4 different muscle sections.

### 2.3. Warner–Bratzler Shear Force

Warner–Bratzler shear force (WBSF) procedures were conducted according to the American Meat Science Association Research Guidelines for Cookery, Sensory Evaluation, and Instrumental Tenderness Measurements of Meat [22]. Roasts were placed on absorbent pads on trays, overwrapped with polyvinylchloride film, and allowed to thaw at 4 ± 2 °C overnight. Before cooking, three 2.54 cm chops per loin were fabricated and weighed. A thermocouple (30-gauge copper and constantan; Omega Engineering, Stamford, CT, USA) was inserted into the geometric center. Chops were cooked on a Cuisinart Griddler (Cuisinart, Stamford, CT, USA) set to 232 °C. A Doric Minitrend 205 monitor (VAS Engineering, San Francisco, CA, USA) was used to monitor the internal temperature. When an internal temperature of 65 °C was reached, the chops were removed from the griddle. The peak temperature averaged 71 °C. The chops were reweighed to determine the cooking loss, then they were chilled for 24 h at 0 ± 2 °C. Six 1.27 cm cores (n = 2/chop) were taken from chops that were parallel to muscle fiber orientation. A Warner–Bratzler shear head on an Instron testing machine (Model 5569; Instron, Canton, MA, USA) with a 100 kg compression load cell was used to shear the cores once they were in the center at a crosshead speed of 250 mm/min. The 6 individual shear values were averaged to derive a value for each chop.

### 2.4. Collagen Content

The remaining meat tissue was trimmed of excess fat and epimysium connective tissue, frozen in liquid nitrogen, pulverized using a Waring blender (Waring Products Division, Hartford, CT, USA), and stored at −80 °C until analysis (approximately 1 month). The protocol from Hill [23] and the Association of Official Analytical Chemists method 990.26 [24] were adapted to determine the hydroxyproline content. Duplicate, pulverized tissue (3 g) was mixed with 12 mL of Ringer’s solution and incubated in a 77 °C water bath for 80 min, with mixing occurring every 10 min. The resulting slurries were centrifuged at 2250× *g* for 12 min at 20 °C to separate the insoluble and soluble fractions. Sulfuric acid (3 mL of concentrated sulfuric acid to the soluble portion; 30 mL of 3.5 M sulfuric acid to the insoluble portion) was added to each fraction and incubated at 105 °C for 16 h. The samples were removed, cooled, diluted with deionized water (250 mL for soluble; 500 mL for insoluble fraction), and filtered with Whatman 541 filter paper (Fisher Scientific, Waltham, MA, USA).

The hydroxyproline concentration was determined following procedures of Bergman and Loxley [25] using a Jasco V-630 spectrophotometer (Jasco Inc., Easton, MD, USA) measuring absorbance at 558 nm. Readings were quantified by a blank, and standard curves were prepared each day of analysis. The collagen content was determined by multiplying the hydroxyproline content of the soluble fraction by 7.25 and the insoluble fraction by 7.52 [26]. Duplicate samples with a coefficient of variation (CV) greater than 15% were re-analyzed.

### 2.5. Gene Expression

Tissue homogenization was performed following the methods previously described [6]. According to the manufacturer’s protocol, RNA extraction and genomic DNA removal were performed using the RNeasy Mini Kit (QIAGEN, Germantown, MD, USA) and TURBO DNA-free Kit (Invitrogen), respectively. The quality of RNA was determined using the TapeStation system (Agilent, Santa Clara, CA, USA). Extracted RNA (10 μL) was reverse transcribed with M-MLV (Invitrogen) according to the manufacturer’s protocol. Real-time RT-PCR primers were designed using NCBI Primer-BLAST and synthesized by Integrated DNA Technologies (Coralville, IA, USA). Melting curves and agarose gel electrophoresis were performed to ensure a single specific PCR product for the target gene. Real-time RT-PCR was performed using Power SybrGreen Master Mix (Invitrogen) and the QuantStudio 6 Pro Real-Time PCR System (Applied Biosystems, Waltham, MA, USA). Each reaction contained 5.0 µL of cDNA, 3.0 µL of nuclease-free water, 1.0 µL of each forward and reverse primer at 10 nmol/L (Supplementary Table S2), and 10 µL of SybrGreen, resting in a total reaction volume of 20 µL. ^Δ^Ct values were determined and used for the ^ΔΔ^Ct value calculation to obtain the relative gene expression [27]. Ribosomal protein S15 (RPS15) mRNA expression was used as the internal control and did not differ between treatment groups (*p* ≥ 0.47).

### 2.6. Statistical Analysis

Data were analyzed using the R programming language in R Studio (version 4.2.1; R Core Team, Vienna, Austria, 2022) on “Spotted Wakerobin” release for macOS, using the packages car [28], emmeans [29], ggpubr [30], lme4 [31], rstatix [32], and tidyverse [33]. Outcomes were analyzed as a one-way ANOVA with maternal diet as the fixed effect. The experimental unit for all variables was the individual animal. Post hoc pairwise comparisons were made using emmeans where appropriate, using the Sidak adjustment for multiple comparisons. Differences were determined to be significant at *p* ≤ 0.05 or a tendency at 0.05 < *p* ≤ 0.10.

## 3. Results

### 3.1. Carcass Traits and Meat Quality

There were no observed effects of poor maternal nutrition during gestation on body weight, LM or STN weight, backfat thickness, or the rib eye area (REA) in the offspring at 282 d of age, as previously reported [21]. In the STN, muscle fiber CSA was less in the OVER and RES rams than in the CON rams (Table 1; *p* = 0.02), with no differences observed between the OVER and RES rams. Longissimus muscle fiber CSA was larger in the RES than CON and OVER rams (Table 1; *p* = 0.002), but no differences were observed between the CON and OVER rams. In the LM, WBSF was less in the OVER rams compared with the CON and RES rams (Table 2; *p* = 0.03). There were no observed effects of maternal diet on thaw or cooking loss (Table 2; *p* ≥ 0.17). There were no observed effects of maternal diet on insoluble, soluble, or total collagen content in the LM (Table 3; *p* ≥ 0.15).

### 3.2. Gene Expression in the Longissimus and Semitendinosus Muscles

The effects of maternal diet on the gene expression of fibrogenic genes in LM are shown in Table 4. In LM, RES rams had 1.5-fold greater expression of *bone morphogenic protein 1 (BMP1)* than the CON (*p* = 0.07) and OVER (*p* = 0.02) rams. There was a tendency for maternal diet to alter the expression of *fibronectin 1* (*FN1*; *p* = 0.07), *lysyl oxidase* (*LOX*; *p* = 0.07), and *collagen 1A1* (*COL1A1*; *p* = 0.08). *Fibronectin 1* expression was 1.4-fold greater in the RES than in OVER (*p* = 0.02). Expression of *LOX* in OVER was downregulated by 1.4-fold compared with the CON rams (*p* = 0.04), and it tended to be downregulated by 1.3-fold compared with the RES rams (*p* = 0.08). Expression of *COL1A1* was 2.0-fold greater in the RES than in CON (*p* = 0.05), and it tended to be 2.2-fold greater in the RES than in OVER (*p* = 0.07). Taken together, there was an overall decrease in fibrogenic gene expression in OVER rams. Myogenic gene expression in the LM was also impacted by dietary treatment. Expression of *paired box y (Pax7)* was 1.8-fold greater in the RES than in OVER (*p* = 0.01). However, there were no observed effects of maternal diet on expression of *myogenic differentiation 1 (MyoD)*, *myogenin*, or *myostatin* (*p* ≥ 0.33). Poor maternal diet during gestation had no observed effects on the expression of adipogenic genes, including *fatty acid binding protein 4* (*FABP4*), *CCAAT/enhancer-binding protein α* (*CEBPα)*, and *peroxisome proliferator-activated receptor γ* (*PPARγ*) in the LM (*p* ≥ 0.18). Because the effects of poor maternal diet may be passed through epigenetic mechanisms, expression of the genes related to epigenetic modifications was investigated in the LM. Poor maternal diet significantly altered the expression of *histone deacetylase 1* (*HDAC1*; *p* = 0.03) and *Lysine acetyltransferase* (*KAT2B*; *p* = 0.03), where the RES rams had 2.8-fold greater *HDAC1* expression than the OVER rams (*p* = 0.04), and the CON rams had 2.0-fold greater *KAT2B* expression than the RES rams (*p* = 0.01). Maternal diet tended to alter the expression of *DNA methyltransferase* (*DNMT1*; *p* = 0.09).

In the STN, the OVER rams had greater *myostatin* than the RES rams, but were not different than the CON rams (Table 5; *p* = 0.02). *Bone morphogenic protein 1* tended to be greater in the RES rams than in the CON rams (*p* = 0.09). The STN in rams from restricted-fed dams tended to have 1.7- and 2-fold greater *COL1A1* and *COL3A1* expression, respectively, than the CON rams (Table 5; *p* = 0.06). There were no observed differences due to diet in other myogenic or adipogenic gene expression in the STN (*p* ≥ 0.16).

## 4. Discussion

A poor maternal diet during gestation altered muscle fiber CSA in the LM and STN, and WBSF in the LM in male offspring at 282 d of age. These phenotypic changes were accompanied by changes in fibrogenic and myogenic gene expression, supporting changes in muscle growth and collagen deposition.

Maternal diet during gestation affects muscle growth. In the current study, restricted nutrition during gestation increased muscle fiber CSA in the LM, while restricted feeding and overfeeding decreased CSA in the STN. The increased muscle fiber CSA in RES offspring LM, despite similar REAs, indicates the presence of fewer muscle fibers, although this was not directly measured in this study. We have previously demonstrated larger muscle fiber CSA in offspring of restricted-fed and overfed ewes at birth, which did not persist to 3 months of age [6]. The secondary/primary fiber ratio was reduced in the LM in offspring of both restricted-fed and overfed ewes, suggesting a reduction or delay in secondary myogenesis [34]. Further, satellite cells, or muscle progenitor cells, isolated from offspring of restricted-fed ewes demonstrated an altered expression of myogenic regulatory factors MyoD and myogenin, suggesting possible delays in prenatal muscle fiber development and growth [35]. Correspondingly, the fetal offspring of ewes restricted-fed during late gestation demonstrated fewer myofibres per mm^2^ in the triceps brachii (TB; [8]). In contrast, muscle fiber CSA in the STN was larger in the CON offspring than in the OVER or RES offspring, despite no significant differences in STN weight relative to body weight [21]. Muscle-specific responses to maternal diet are not uncommon [21,36,37]. Differences in muscle metabolism, structure, and use may all contribute to these differential responses, which may also vary throughout an animal’s life due to the plasticity of muscle. Muscle fiber number and CSA are positively correlated with muscle mass and the lean meat percentage [38]. Differences in muscle fiber CSA as a result of maternal diet during gestation may impact the lean meat percentage, and therefore, meat quality in a muscle-specific manner, and require additional research.

Maternal diet during gestation may also affect meat quality. The Warner–Bratzler shear force was less in the LM of OVER rams, indicating increased tenderness. Among other factors, muscle fiber size, collagen content, and fat content can affect tenderness [39,40,41]. A high-protein diet (14%) in cows during early gestation reduced the WBSF in the offspring STN but not the LM, which is relative to the offspring of dams fed a low-protein diet [42]. In contrast, a high-energy diet during gestation did not alter backfat thickness or the WBSF in male or female pig offspring [43]. Differences in the WBSF have also been identified as a result of maternal nutrient restriction. For example, a reduced WBSF was demonstrated in the STN of mature female offspring of ewes that were nutrient restricted from day 30 to 143 of gestation [44]. Similarly, a 50% nutrient restriction during gestation resulted in a decreased WBSF in offspring compared with offspring of moderately (75% NRC) and non-nutrient (100% NRC)-restricted dams [45]. Further, LM tenderness was increased after both 3 and 14 days of aging in the offspring of cows that were fed greater amounts of protein compared with the offspring of cows that were fed reduced protein during gestation [46].

In the current work, poor maternal nutrition during gestation did not alter the collagen content in the LM of offspring. Similar to our current findings, there were no differences in the total or insoluble collagen contents in the LM of steers from dams that were nutrient restricted during late gestation compared with the control steers [45], or in the offspring of dams fed a high- or low-protein diet during late gestation [46]. In contrast, the collagen concentration was increased in the LM and STN of 2.5-year-old rams born to ewes that were fed an obesogenic diet (150% NRC) during gestation [10]. These rams also had greater expression of *LOX*, *lysyl hydroxylase-2b*, and *prolyl-4 hydroxylase*, suggesting increased collagen crosslinking. The discrepancies between these studies may be due to the difference in age; our animals were approximately 10 months of age at sample collection, compared with 2.5 years of age. Changes in collagen deposition and crosslinking may become more apparent with age. While we did not observe effects of poor maternal diet on the collagen content of the offspring, LM, the RES rams had greater LM expression of *BMP1*, *FN1*, *LOX*, and *COL1A1* than the OVER rams. Further, in the STN, the RES rams had greater *COL1A1* and *COL3A1* gene expression. Greater *COL1A1* expression in both LM and STN suggests the presence of more collagen in these muscles in RES rams than in CON or OVER rams. As collagen matures, intermolecular crosslinks form, decreasing collagen solubility [47]. Increased crosslinking is directly related to reduced tenderness and increased shear force [48]. Bone morphogenic protein 1 activates the LOX pro-protein, converting it to active LOX [49,50]. Activated LOX enzyme is essential for collagen crosslink formation. Thus, an increased expression of *BMP1* and *LOX* in RES offspring LM suggests the possibility of greater collagen crosslinking and a subsequent decrease in tenderness. Similarly, fetal offspring of restricted-fed cows demonstrated an increased expression of *LOX* and *BMP1* in infraspinatus muscle; however, no significant differences in total, soluble, or insoluble collagen were detected, and the WSBF was not quantified [36]. In the current work, the WBSF was decreased in the offspring of overfed ewes compared with control-fed and restricted-fed ewes. This may be related to the reduction in *LOX* expression in the LM, indicating less collagen crosslinking despite no observed differences in insoluble, soluble, or total collagen content. These discrepancies in gene expression and collagen content may be due to methodology, since the methods for measuring soluble and insoluble collagen content are not as sensitive as the real-time PCR. The changes in gene transcription may not result in changes in collagen content that are sufficient to be measured with the methods employed. It is also possible that as these animals age, the phenotype may become more apparent, with greater collagen deposition due to potentially sustained increased gene expression, as seen in other studies [10].

In the current work, we did not identify any changes in the muscle lipid content or gene expression of *CEBPα*, *FABP4*, or *PPARγ*, moderators of adipogenesis [51,52,53], in STN or LM. In contrast, previous work demonstrated that mature offspring of ewes that were nutrient restricted from day 30 to 143 of gestation exhibited an increased lipid content in the supraspinatus of female lambs [44]. Overfeeding during gestation increased the muscle lipid content in offspring at 3 months of age, while restricted feeding during gestation decreased the muscle lipid content in offspring at the same age [6]. The differences between studies may be due to differences in the timing and (or) severity of the nutritional restriction, the muscles used for analysis, or the postnatal management of the animals (e.g., bottle-fed or a suckling ewe).

Differences in muscle growth due to poor maternal nutrition during gestation may be passed to multiple generations of offspring through epigenetic mechanisms [54,55]. The offspring of overfed ewes demonstrated reduced *HDAC1* gene expression. When bound to MyoD, *HDAC1* can result in deacetylation that inhibits muscle-specific genes such as myosin heavy chain [56]. Expression of *HDAC1* gradually decreases naturally during myoblast differentiation [56]. Increased expression of *DNMT1* in RES rams compared with the OVER rams may indicate increased DNA methylation in these animals. Specifically, DNMT1 is responsible for the maintenance of DNA methylation patterns in muscle, and a loss of DNMT1 results in reduced muscle mass [57] and the number of muscle satellite cells [58] in mice. As *Pax7* specifies satellite cell fate [59], greater expression of *Pax7* in the RES than the OVER offspring in the current study could be driven by the alteration in *DNMT1* expression, supporting the observed greater muscle fiber CSA in RES offspring. Further, expression of *KAT2B*, a lysine acetyltransferase, was reduced in RES compared with the CON offspring. Lysine acetyltransferase 2B is associated with histone acetyl transferase activity, particularly at the H3K9 sites, and is highly expressed during myoblast proliferation [60]. In beef [61] and dairy [62] cattle, *KAT2B* serves as a molecular marker to improve the selection for growth performance. As previously discussed, the increased muscle fiber CSA in RES offspring, despite having a similar REA, indicates the presence of fewer muscle fibers. It is possible that the reduced expression of *KAT2B* in RES rams may lead to the greater CSA observed as a compensatory mechanism to maintain muscle mass. Together, these changes in epigenetic markers in muscle may be a mechanism by which these effects on muscle mass are passed to subsequent generations and, as such, require additional research.

In summary, poor maternal nutrition during gestation decreased STN muscle fiber CSA in RES and OVER offspring, increased LM muscle fiber CSA in RES offspring, decreased LM WBSF in OVER offspring, and altered gene expression related to collagen deposition and crosslinking, as well as myogenesis and epigenetic markers in a diet- and muscle-specific manner. Further research investigating how dietary changes during gestation may affect muscle growth and meat quality is required to better understand potential management paradigms so as to improve growth and product quality.

## Figures and Tables

**Table 1:** Carcass traits in ram muscle at 282 days of age.

	Treatment				
Item	CON	RES	OVER	SEM	*p*-Value ^2^
LM ^1^, g/kg BW	11.50	11.90	11.92	0.45	0.76
Backfat ^1^, cm	0.45	0.48	0.49	0.03	0.66
REA ^1^, cm^2^	38.00	35.20	38.80	1.75	0.24
LM CSA, µm^2^	2497 ^a^	2991 ^b^	2719 ^a^	93	0.002
STN CSA, µm^2^	5674 ^a^	4953 ^b^	4893 ^b^	177	0.007

Abbreviations: CON = rams from control-fed ewes (100% NRC, n = 12); OVER = rams from overfed ewes (140% NRC, n = 13); RES = rams from restricted-fed ewes (60% NRC, n = 18); LM = longissimus muscle; REA = rib eye area; CSA = cross-sectional area; STN = semitendinosus. ^1^ Reported in [21] ^2^ Means with different superscripts (a,b) differ (*p* ≤ 0.05).

**Table 2:** Cooking traits in ram longissimus muscle.

	Treatment				
Item	CON	RES	OVER	SEM	*p*-Value ^1^
Initial temperature, °C	21.34	21.11	20.99	0.45	0.88
Final temperature, °C	70.75	70.92	70.94	0.86	0.99
Thaw loss, %	8.99	10.4	9.26	0.58	0.17
Cooking loss, %	19.83	19.73	18.50	0.81	0.46
WBSF, kg	2.19 ^a^	2.10 ^a^	1.73 ^b^	0.12	0.03

Abbreviations: CON = rams (282 d of age) from control-fed ewes (100% NRC, n = 12); OVER = rams (282 d of age) from overfed ewes (140% NRC, n = 13); RES = rams (282 d of age) from restricted-fed ewes (60% NRC, n = 18); WBSF = Warner–Bratzler shear force. ^1^ Means with different superscripts (a,b) differ (*p* ≤ 0.05).

**Table 3:** Collagen content in ram longissimus muscle.

	Treatment				
Item	CON	RES	OVER	SEM	*p*-Value
Insoluble collagen, mg/g	11.5	11.9	9.46	1.01	0.19
Soluble collagen, mg/g	2.00	1.84	1.82	0.19	0.78
Total collagen, mg/g	13.5	14.2	11.3	1.11	0.15

Abbreviations: CON = rams (282 d of age) from control-fed ewes (100% NRC, n = 12); OVER = rams (282 d of age) from overfed ewes (140% NRC, n = 13); RES = rams (282 d of age) from restricted-fed ewes (60% NRC, n = 18).

**Table 4:** Gene expression ^1^ in the ram longissimus muscle.

		Treatment				
Gene Category	Gene	CON	RES	OVER	SEM	*p*-Value ^2^
Adipogenesis	CEBPα	1.29	1.19	1.57	0.29	0.67
	FABP4	1.47	2.20	2.17	0.51	0.66
	PPARγ	1.11	1.37	1.16	0.16	0.18
Fibrogenesis	COL1A1	1.35 ^x^	2.68 ^y^	1.22 ^x^	0.34	0.08
	COL3A1	1.38	2.89	1.40	0.37	0.19
	CST3	1.12	1.16	0.81	0.13	0.19
	FN1	1.15 ^xy^	1.28 ^x^	0.92 ^y^	0.18	0.07
	LOX	1.23 ^x^	1.09 ^x^	0.86 ^y^	0.19	0.07
	BMP1	1.09 ^a^	1.60 ^b^	1.05 ^a^	0.15	0.01
Myogenesis	MSTN	1.22	1.13	1.04	0.21	0.78
	MYOD1	1.28	1.10	1.25	0.25	0.79
	MYOG	1.25	1.11	1.00	0.23	0.33
	Pax7	1.36 ^ab^	1.50 ^a^	0.83 ^b^	0.31	0.02
Epigenetic	ASH1L	1.38	1.77	1.78	0.39	0.97
	DNMT1	1.23 ^xy^	1.50 ^x^	1.02 ^y^	0.30	0.09
	DNMT3B	1.33	1.23	0.92	0.27	0.20
	EHMT1	1.47	1.02	1.03	0.33	0.55
	EHMT2	1.35	1.06	0.99	0.30	0.46
	HAT1	1.31	0.99	1.67	0.35	0.13
	HDAC1	1.26 ^a^	1.82 ^a^	0.64 ^b^	0.31	0.03
	HDAC2	1.05	1.13	1.18	0.11	0.81
	HDAC3	1.05	1.04	1.03	0.09	0.99
	HDAC4	1.17	1.13	1.25	0.24	0.93
	HDAC5	1.06	1.19	1.03	0.16	0.90
	HDAC6	1.27	1.92	0.82	0.38	0.19
	HDAC8	1.16	1.02	0.99	0.19	0.68
	HDAC11	1.16	1.34	1.47	0.26	0.63
	KAT2B	1.17 ^a^	0.58 ^b^	0.76 ^ab^	0.13	0.01
	KAT6A	1.14	1.65	1.32	0.31	0.88
	KAT8	1.06	0.86	0.98	0.10	0.32
	SETDB2	1.69	1.33	0.76	0.30	0.47
	SIRT1	1.14	1.16	1.34	0.24	0.14
	TET2	2.01	1.36	1.31	0.50	0.98

Abbreviations: CON = rams from control-fed ewes (100% NRC, n = 12); OVER = rams from overfed ewes (140% NRC, n = 13); RES = rams from restricted-fed ewes (60% NRC, n = 18); ASH1L = ASH1-like histone lysine methyltransferase; BMP1 = Bone morphogenic protein; CEBPα = CCAAT/enhancer-binding protein α; COL1A1 = Collagen A1A; COL3A1 = Collagen A3A; CST3 = Cystatin-c; DNMT = DNA methyltransferase; EHMT = Euchromatic histone lysine methyltransferase; FABP4 = Fatty acid binding protein 4; FN1 = Fibronectin; HDAC = Histone deacetylase; KAT = Lysine acetyltransferase; LOX = Lysyl oxidase; MSTN = Myostatin; MYOD1 = Myogenic differentiation 1; MYOG = Myogenin; PAX7 = Paired box protein; PPARγ = Peroxisome proliferator-activated receptor γ; SETDB2 = SET domain bifurcated histone lysine methyltransferase 2; SIRT1 = Sirtuin 1; TET2 = Ten eleven translocation enzyme-2. ^1^ Relative to CON, mean. ^2^ Means with different superscripts (a,b) differ (*p* ≤ 0.05); means with different superscripts (x,y) tend to differ (0.05 < *p* ≤ 0.10).

**Table 5:** Gene expression ^1^ in ram semitendinosus muscle.

		Treatment				
Gene Category	Gene	CON	RES	OVER	SEM	*p*-Value ^2^
Adipogenesis	CEBPα	1.14	1.01	1.61	0.18	0.44
	FABP4	1.81	2.31	2.43	0.55	0.48
	PPARγ	1.15	1.47	1.81	0.22	0.16
Fibrogenesis	COL1A1	1.38 ^x^	2.29 ^y^	1.90 ^xy^	0.31	0.06
	COL3A1	1.19 ^x^	2.43 ^y^	2.36 ^xy^	0.34	0.06
	CST3	1.09	1.02	1.46	0.16	0.33
	FN1	1.83	1.95	1.71	0.37	0.49
	LOX	1.34	1.50	2.50	0.30	0.13
	BMP1	1.25 ^x^	1.83 ^y^	1.60 ^xy^	0.23	0.09
Myogenesis	MSTN	1.15 ^ab^	1.00 ^a^	1.84 ^b^	0.19	0.02
	MYOD1	1.16	0.98	1.48	0.19	0.40
	MYOG	1.09	0.93	1.17	0.17	0.50
	Pax7	Undet	Undet	Undet	N/A	N/A

Abbreviations: CON = rams from control-fed ewes (100% NRC, n = 12); OVER = rams from overfed ewes (140% NRC, n = 13); RES = rams from restricted-fed ewes (60% NRC, n = 18); BMP1 = Bone morphogenic protein; CEBPα = CCAAT/enhancer-binding protein α; COL1A1 = Collagen A1A; COL3A1 = Collagen A3A; CST3 = Cystatin-c; FABP4 = Fatty acid binding protein 4; FN1 = Fibronectin; LOX = Lysyl oxidase; MSTN = Myostatin; MYOD1 = Myogenic differentiation 1; MYOG = Myogenin; Pax7 = Paired box protein; PPARγ = Peroxisome proliferator-activated receptor γ; Undet = undetectable; N/A = not applicable. ^1^ Relative to CON, mean. ^2^ Means with different superscripts (a,b) differ (*p* ≤ 0.05); means with different superscripts (x,y) tend to differ (0.05 < *p* ≤ 0.10).

## Data Availability

The raw data supporting the conclusions of this article will be made available by the authors upon request.

## Supplementary Materials

The following supporting information can be downloaded at: https://www.mdpi.com/article/10.3390/ani16030486/s1, Table S1: Chemical composition of F0 ewe and F1 offspring diets. Table S2: Primers for gene expression.
