# Supplementary material for: Poor Maternal Diet During Gestation Alters Offspring Muscle Morphometrics, Collagen Gene Expression, and Meat Tenderness in Sheep
Source: Animals (Basel). 2026 Feb 4;16(3):486. doi: 10.3390/ani16030486 (PMC12896928; doi:10.3390/ani16030486)
Supplement: Supplementary file 1 [file animals-16-00486-s001.zip › animals-4079086-supplementary.pdf]

Table S1. Chemical composition of F0 ewe and F1 offspring diets[1]

Nutrient Analysis[2]	F0 Complete Pellet[3]	F1 Creep Feed[4]	F1 Grower Feed[5]	F1 Complete Pellet[6]
Moisture, %	11.96	10.80	13.50	9.45
Dry Matter, %	88.01	89.20	86.60	90.57
Crude Protein, %	13.38	21.20	18.50	18.48
Adjusted Crude Protein, %	13.38	21.20	18.50	18.48
ADF, %	28.19	11.70	11.70	25.95
aNDF, %	38.52	18.80	24.70	40.28
TDN, %	74.75	81.00	79.00	74.33
DE, Mcal/kg	2.87	3.37	3.26	2.80
NE_L, Mcal/kg	1.76	1.91	1.87	1.75
NE_M, Mcal/kg	1.78	2.00	1.94	1.77
NE_G, Mcal/kg	1.16	1.34	1.30	1.15
Calcium, %	1.34	0.75	1.45	1.42
Phosphorus, %	0.42	0.63	0.68	0.54
Magnesium, %	0.25	0.28	0.32	0.34
Potassium, %	1.19	1.58	1.00	2.16
Sodium, %	0.18	0.25	0.35	0.51
Iron, mg/kg	641.75	394.00	270.00	358.50
Zinc, mg/kg	128.33	316.00	187.00	103.17
Copper, mg/kg	13.50	6.00	4.00	11.67
Manganese, mg/kg	82.25	158.00	125.00	86.33
Molybdenum, mg/kg	3.99	12.50	0.50	5.83
Sulfur, %	0.32	0.45	0.42	0.29

[1]From [21]
[2]Nutrient analyses were performed by Dairy One, Inc. (Ithaca, NY).
[3]Values are presented as an average analysis of 9 bags of grain for entire experiment.
[4]Offspring were fed Creep Feed until d 120 of age.
[5]Offspring were fed Grower Feed from d 121 to 153 of age.
[6]Offspring were fed Complete Feed from d 154 to 282 of age. Values are presented as an average analysis of four deliveries.

Table S2. Primer sequences

Gene	Primer sequences (5' → 3')	Amplicon length	Reference
ASH1L			
Forward	TCGGCCTCTGACACGAGAAA	282	XM_027976130.1
Reverse	TTTCCCGATTCCACTTGCGA		
BMP1			
Forward	GGCAAGTTCTGTGGCTCTGA	137	XM_042243151.1
Reverse	GAGCACTCGTCCTTGTCTGA		
CEBPa			
Forward	CATGCCGGGAGGACTTTAGC	215	NM_001308574.1
Reverse	GTCGATGGACGTCTCGTGTT		
COL1A1			
Forward	ACGTGATCTGCGACGAACTT	99	XM_027974705.2
Reverse	GGTCCGTGGTTGATTCCTGG		
COL3A1			
Forward	CAAAGGTGAAATGGGTCCTGC	129	XM_004004514.5
Reverse	TTCTTACCGGGTTCACCTGC		
CST3			
Forward	GTCAGCGAGTTCAACAAGCG	204	NM_001280713.1
Reverse	GGAGCACAGCTTTTCCCTCTT		
DNMT1			
Forward	GCGGTACAACTACCACGACA	133	NM_001009473.1
Reverse	CAGGCACCAGGGAATAAGGG		
DNMT3B			
Forward	AGTACCTCATCGGGAGCAGA	119	XM_027977073.1
Reverse	AAATGAGCACCGTGTTTGGC		
EHMT1			
Forward	CAGACAGCTTGCTTGCCTTT	121	XM_027966020.1
Reverse	TCACCGTCCTCTGCTTCAAC		
EHMT2			
Forward	AGTGTAACCAGGCATGCTCC	168	XM_027958814.1
Reverse	TCAGTTCTCCGACATACTCGC		
FABP4			
Forward	ATGAAAGAAGTGGGTGTGGGC	253	NM_001114667.1
Reverse	TGGTGGTTGATTTCCCATCCC		
FN1			
Forward	CCCAACTCCCTGCTGGTATC	243	XM_004004910.5
Reverse	GAGAGCTTCTTGTCCTGTCTT		

HAT1			
Forward	ACACTCTTTGCGACCGTAGG	200	XM_004004594.3
Reverse	TTGGATGGATCTTCCGCTGT		
HDAC1			
Forward	TTCAACGTCGGTGAGGACTG	162	XM_004005023.3
Reverse	TGCCTCGGACTTCTTTGCAT		
HDAC2			
Forward	CCATGGCGTACAGTCAAGGA	129	XM_004011189.3
Reverse	GCAAGTTATGGGTCATGCGG		
HDAC3			
Forward	GAGGACACGGGGAATGTGTT	190	XM_004008901.4
Reverse	GTGAAGTCCGGGGCAAAGTA		
HDAC4			
Forward	TCGCTACTGGTACGGGAAGA	144	XM_027967041.1
Reverse	GCGCAATTTCAGGTTGGGTT		
HDAC5			
Forward	GTCGAAGTCAAAGGAGCCCA	168	XM_027974446.1
Reverse	CTGTCATAGGGCCCAAGCAA		
HDAC6			
Forward	GGAAGCCACGGGGAGGA	101	XM_027962812.1
Reverse	TTGTGCCTACTCCTTCGCTC		
HDAC8			
Forward	AGCTGTAGGAGGGGCTACAA	170	XM_004022188.4
Reverse	CAAATTTCCGTCGCAACCGT		
HDAC11			
Forward	ACGGAGGATGACGAGTACCT	186	XM_027957834.1
Reverse	ACGGACTATCCGGAACACCA		
KAT2B			
Forward	TCCCGTACACGGAGTTCTCT	189	XM_027962092.1
Reverse	CTCTTCCACTCGGCTTCCAG		
KAT6A			
Forward	TCAGATTGGCCCACAGACAAT	133	XM_027962617.1
Reverse	GCAGCGCTTGTTCTTGGATG		
KAT8			
Forward	ACTCGCAACCAAAAACGCAA	116	XM_027961920.1
Reverse	TTCACCTTGGTGATCGCCTC		
LOX			
Forward	GTGACTACGGCTACCACAGG	278	XM_027969995.2
Reverse	TTGGCTTGCTTTCTAATACGGTG		

MSTN			
Forward	TCCACTCCGGGAACTGATTG	133	NM_001009428.3
Reverse	TGCTAGAAGATCAGACTCCGTG		
MyoD1			
Forward	CGCCCAAAAGATTGCGCTTA	104	NM_001009390.1
Reverse	GGCGGAAACACAACAGTTCC		
MyoG			
Forward	ACCTCACTTCTATGACGGGGA	188	NM_001174109.1
Reverse	TCCGCTTGCACACCTTACAC		
Pax7			
Forward	CAAGATCGTGGAGATGGCCC	296	XM_027965643.2
Reverse	TCGAACTCACTAAACCTGAGGG		
PPARγ			
Forward	TATTCTCAGTGGAGACCGCC	160	NM_001100921.1
Reverse	CTGCCTGAGGTCCGTCATTT		
RPS15			
Forward	GGCGCAGAACGAAAAATCCT	238	XM_015096022.3
Reverse	GCATCTTTCTTGGCCTTGCG		
SETDB2			
Forward	TAGAACTGGAAGCACGTCCG	295	XM_004012076.4
Reverse	TGGTTTGAGCTTGAGTCCTTTCT		
SIRT1			
Forward	CAAGACCTCGGATAGGTCCA	116	XM_015104377.2
Reverse	ATCCAATTCAGGCGGAGGAA		
TET2			
Forward	TGAAACCGGAACCAATCTGT	278	XM_027970835.1
Reverse	TAAAGTGCAGACCACTGTGC		

Abbreviations: ASH1L = ASH1-like histone lysine methyltransferase; BMP1 = Bone-morphogenic protein; CEBPα = CCAAT/enhancer-binding protein α; COL1A1 = Collagen A1A; COL3A1 = Collagen A3A; CST3 = Cystatin-c; DNMT = DNA methyltransferase; EHMT = Euchromatic histone lysine methyltransferase; FABP4 = Fatty acid binding protein 4; FN1 = Fibronectin; HDAC = Histone deacetylase; KAT = Lysine acetyltransferase; LOX = Lysyl oxidase; MSTN = Myostatin; MyoD1 = Myogenic differentiation 1; MyoG = Myogenin; PAX7 = Paired box protein; PPARγ = Peroxisome proliferator-activated receptor γ; RPS15 = Ribosomal protein S15; SETDB2 = SET domain bifurcated histone lysine methyltransferase 2; SIRT1 = Sirtuin 1; TET2 = Ten Eleven Translocation Enzyme-2.
